# Validation of Single Centre Pre-Mobile Atrial Fibrillation Apps for Continuous Monitoring of Atrial Fibrillation in a Real-World Setting: Pilot Cohort Study

**DOI:** 10.2196/14909

**Published:** 2019-12-03

**Authors:** Hui Zhang, Jie Zhang, Hong-Bao Li, Yi-Xin Chen, Bin Yang, Yu-Tao Guo, Yun-Dai Chen

**Affiliations:** 1 Department of Cardiology Chinese PLA General Hospital Beijing China; 2 Huawei Device Co, Ltd Shenzhen China

**Keywords:** atrial fibrillation, photoplethysmography, continuous detection, accuracy, smartphone, smart band, algorithm

## Abstract

**Background:**

Atrial fibrillation is the most common recurrent arrhythmia in clinical practice, with most clinical events occurring outside the hospital. Low detection and nonadherence to guidelines are the primary obstacles to atrial fibrillation management. Photoplethysmography is a novel technology developed for atrial fibrillation screening. However, there has been limited validation of photoplethysmography-based smart devices for the detection of atrial fibrillation and its underlying clinical factors impacting detection.

**Objective:**

This study aimed to explore the feasibility of photoplethysmography-based smart devices for the detection of atrial fibrillation in real-world settings.

**Methods:**

Subjects aged ≥18 years (n=361) were recruited from September 14 to October 16, 2018, for screening of atrial fibrillation with active measurement, initiated by the users, using photoplethysmography-based smart wearable devices (ie, a smart band or smart watches). Of these, 200 subjects were also automatically and periodically monitored for 14 days with a smart band. The baseline diagnosis of “suspected” atrial fibrillation was confirmed by electrocardiogram and physical examination. The sensitivity and accuracy of photoplethysmography-based smart devices for monitoring atrial fibrillation were evaluated.

**Results:**

A total of 2353 active measurement signals and 23,864 periodic measurement signals were recorded. Eleven subjects were confirmed to have persistent atrial fibrillation, and 20 were confirmed to have paroxysmal atrial fibrillation. Smart devices demonstrated >91% predictive ability for atrial fibrillation. The sensitivity and specificity of devices in detecting atrial fibrillation among active recording of the 361 subjects were 100% and about 99%, respectively. For subjects with persistent atrial fibrillation, 127 (97.0%) active measurements and 2240 (99.2%) periodic measurements were identified as atrial fibrillation by the algorithm. For subjects with paroxysmal atrial fibrillation, 36 (17%) active measurements and 717 (19.8%) periodic measurements were identified as atrial fibrillation by the algorithm. All persistent atrial fibrillation cases could be detected as “atrial fibrillation episodes” by the photoplethysmography algorithm on the first monitoring day, while 14 (70%) patients with paroxysmal atrial fibrillation demonstrated “atrial fibrillation episodes” within the first 6 days. The average time to detect paroxysmal atrial fibrillation was 2 days (interquartile range: 1.25-5.75) by active measurement and 1 day (interquartile range: 1.00-2.00) by periodic measurement (*P*=.10). The first detection time of atrial fibrillation burden of <50% per 24 hours was 4 days by active measurement and 2 days by periodic measurementThe first detection time of atrial fibrillation burden of >50% per 24 hours was 1 day for both active and periodic measurements (active measurement: *P*=.02, periodic measurement: *P*=.03).

**Conclusions:**

Photoplethysmography-based smart devices demonstrated good atrial fibrillation predictive ability in both active and periodic measurements. However, atrial fibrillation type could impact detection, resulting in increased monitoring time.

**Trial Registration:**

Chinese Clinical Trial Registry of the International Clinical Trials Registry Platform of the World Health Organization ChiCTR-OOC-17014138; http://www.chictr.org.cn/showprojen.aspx?proj=24191.

## Introduction

Atrial fibrillation (AF) is the most common recurrent arrhythmia in clinical practice, occurring in 1%-2% of the general population [1]. Two epidemiological studies found an AF prevalence rate of approximately 0.7% in the Chinese population nearly a decade ago [2,3]. Other studies found that the prevalence of AF increased 20-fold and that of AF-related stroke increased 13-fold in the southwest of China from 2001 to 2012 [4]. As such, it was estimated that there were 10 million patients with AF in China in 2016 [5]. When identified clinically, AF is asymptomatic in at least one-third of patients [6]. Some patients experience very brief episodes, associated with vague symptoms. Therefore, it is possible that these epidemiological numbers are underestimated, as many patients with asymptomatic or paroxysm AF could be undetected [7,8]. However, the prevalence of AF is expected to increase significantly in the next 30-50 years due to an ageing population. The prevalence of AF in Korea is expected to be 5.81% in 2060, while its prevalence in Taiwan is estimated to be 4.01% in 2050 [9,10]. Furthermore, AF is an important risk factor for stroke, increasing the stroke risk five-fold, and accounts for nearly one-third of all stroke cases [11-13]. Thus, AF is likely to become a significant public health issue in the future. Early diagnosis and anticoagulation therapy could reduce the number of AF-related strokes by approximately two-thirds [14], making it particularly important to effectively detect AF as early as possible.

However, paroxysmal AF could be underdiagnosed because there is possibly no onset in the hospital [15]. Mobile health could provide a promising solution [16]. The popularity and high-speed updating capabilities of smartphone apps and wearable fitness trackers have made continuous self-monitoring of health indicators possible in real-world settings. Patients and clinicians can utilize these technologies for proactive health care. For example, people can use smartphone apps and smart bands to count steps, measure sleep time, and continuously monitor heart rate/rhythm.

Photoplethysmography is an optical method to measure changes in tissue blood volume through the skin capillary bed, which can be performed using a smartphone in conjunction with a smart band/watch without any additional peripherals [17,18]. Smartphones and wearable devices have leveraged optical detection of photoplethysmography signals to track heart rate/rhythm from the finger and wrist, achieving high sensitivity and specificity for AF detection [19,20]. Thus far, most studies examining this method of AF detection have used single-point tests. Our previous study confirmed the feasibility of single-point photoplethysmography measurement of smartphones for AF detecting too [21]. A real-world study of patients with AF outside hospital context is limited. The application of photoplethysmography-based smart devices in continuously and automatically monitoring AF risk is unknown. The objectives of this study were to validate the accuracy and sensitivity of continuous detection of AF with photoplethysmography-based smart devices and to investigate the underlying clinical factors impacting the detection.

## Methods

### Study Population

Subjects for this pilot study were recruited from the community and the outpatient department of the Chinese PLA General Hospital from September 14, 2018, to October 16, 2018. We included adult participants aged ≥18 years who were able to provide informed consent and were willing to wear the smart wearable device. Individuals were excluded from the study due to the presence of a pacemaker or implantable defibrillator or if they were unable to use smart wearable devices.

### Study Procedures

Three types of smart wearable devices were used in this study. These included the Huawei WatchGT [22], the Honor Watch [23], and the Honor Band4 [24]. The matching smartphone app was used (Honor9; Huawei Device Co, Ltd, Shenzhen, China). The Huawei smart wearing devices were freely provided for the subjects.

All subjects received active measurement with smart band/watch upon enrollment. Measurement with the devices was taken immediately following doctor diagnosis, and each participant used two of three devices. If the initial two measurements failed, additional measurements were taken. Participants were helped with band/watch placement and instructed to keep their arms still by resting them on a table. The photoplethysmography signal was record for 45 seconds, and a result was provided to detect the single-point heart rhythm.

After active measurement, each of the 200 participants who owned Huawei smart phones were provided with an Honor Band4 to continuously monitor heart rate/rhythm during a 14-day period. Each participant was given assistance in downloading the app to his/her smartphone by a member of the study team. Participants were also trained on how to use the device. They were instructed to actively take two measurements per day and wear the smart band as long as possible in order to record the most automatic periodic measurement data possible. The periodic measurement was automatically taken every 10 minutes, irrespective of whether the subject walked or rested, and 60-second photoplethysmography signals were continuously collected. However, the algorithm was first performed to identify whether the signal quality was good enough to do further analysis. Thereafter, the photoplethysmography algorithm was carried out to screen irregular pulse rhythm. At least a 30-second AF was needed to meet the definition of AF.

This was a single-center pilot study of AF screening and part of the pre-mAFA II registry. The pre-mAFA studies examined mobile health technology for improved screening, patient involvement, and optimization of integrated AF care. The Medical Ethics Committee of the Chinese PLA General Hospital and the China Food and Drug Administration approved this study protocol (approval number: S2017-105-02). Further, this study was registered in the Chinese Clinical Trial Registry, which was part of the International Clinical Trials Registry Platform of the World Health Organization (ChiCTR-OOC-17014138).

### Atrial Fibrillation Detection and Confirmation

AF diagnoses were independently confirmed with medical history, physical examination, and electrocardiogram by two doctors. Patient data such as medical history, physical examination, and electrocardiogram were collected when subjects were enrolled. Doctors used a dual earpiece stethoscope to confirm cardiac rhythm of the same subject. If the doctors disagreed on the diagnosis obtained via the dual earpiece stethoscope and pulse check, the subject was further exposed to a 12-lead electrocardiogram that was examined independently by two additional doctors.

Paroxysmal AF was defined as self-terminating, in most cases, within 48 hours. Some AF paroxysms might continue for up to 7 days. AF episodes that were cardioverted within 7 days were considered to be paroxysmal [25].

Persistent AF was defined as AF that lasted longer than 7 days, including episodes that were terminated by cardioversion, either with drugs or by direct current cardioversion, after 7 days or more [25].

### Photoplethysmography Algorithm

One of the efficient machine learning methods, boosting, was used to train the model to screen AF. Sensitive features extracted from the waveforms and the peak-to-peak intervals of the photoplethysmography were utilized in the model. The peak-to-peak intervals of photoplethysmography were uniform for sinus rhythm data but chaotic for AF episodes. For example, the variance, entropy, etc, derived from the peak-to-peak intervals were fluctuating for AF episodes (Multimedia Appendices 1 and 2). Three smart devices were used to investigate if the layout of the photoplethysmography sensor position influences the accuracy of photoplethysmography algorithm to detect AF.

### Statistical Analyses

Continuous variables were tested for normality by the Kolmogorov-Smirnov test. Data with normal distributions were presented as mean (SD). Data with non-normal distributions were analyzed using a Mann-Whitney U test and were presented as median (interquartile range [IQR]). Categorical variables were analyzed using Pearson chi-square test or Fisher exact test.

Sensitivity and specificity were calculated by periodic interpretation of smart wearable devices compared with physician diagnoses. Kappa coefficients were assessed for diagnostic agreement, which was performed using MedCalc 12.6.1.0 (MedCalc Software BVBA, Ostend, Belgium). Excellent agreement was defined as a kappa coefficient >0.80.

A two-sided *P* value<.05 was considered statistically significant. The 95% CIs were calculated, and statistical analysis was performed using IBM SPSS Statistics, version 25.0 (SPSS Inc, Chicago, Illinois).

## Results

### Baseline Characteristics and Atrial Fibrillation Diagnoses

There were a total of 361 subjects (median age 50 years; IQR=36-62 years); 178 (49.3%) were female, excluding 12 subjects with poor pulse signals. There were 20 diagnoses of paroxysmal AF, 11 of persistent AF, and 330 of sinus rhythm confirmed by doctors via medical history, physical examination, and electrocardiogram upon enrollment.

### Active Measurement by Photoplethysmography

**Figure 1:**
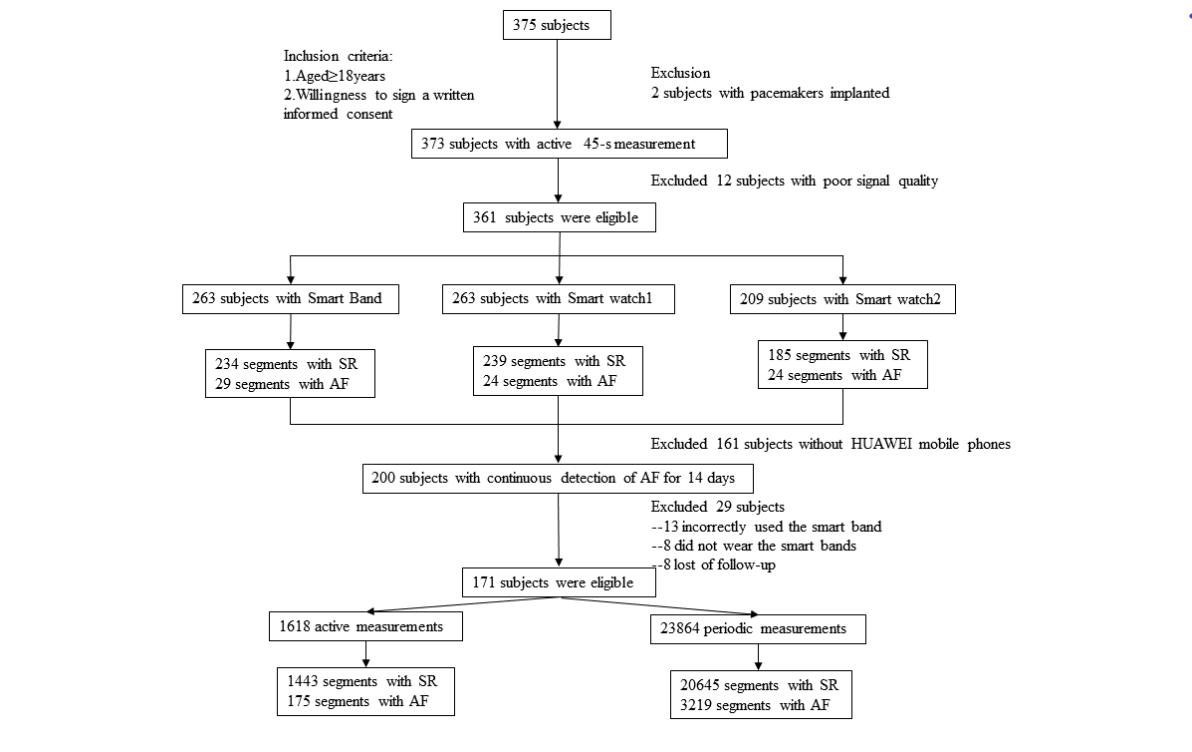
Participant flow diagram of the study. AF: atrial fibrillation，SR: sinus rhythm.

All 361 subjects received a 45-s photoplethysmography active measurement with at least two kinds of smart wearable devices, with a total of 735 valid pulse waveform recordings. Among the 735 valid photoplethysmography signals, 77 signals were classified as AF and 658 were classified as sinus rhythm (Figure 1).

The sensitivity of all of three smart devices with the 45-s photoplethysmography active measurement in predicting AF was 100%, the specificity ranged from 98.93% to 99.16%, the positive predictive value ranged from 91.67% to 93.10%, and the negative predictive value was 100%. Three kinds of smart wearable devices exhibited kappa coefficients ranging from 0.95 to 0.96 to detect AF against the reference standard in the single-point heart rhythm detection. There was no significant difference in further statistical analysis of the results from different smart devices compared with the diagnosis of the doctors (*P*=.97; Table 1).

**Table 1:** Detailed diagnostic performance of the photoplethysmography algorithm for atrial fibrillation screening in different smart devices.

Index	Smart band (n=263)	Smart watch 1 (n=263)	Smart watch 2 (n=209)
Sensitivity, % (95% CI)	100 (87.23-100)	100 (85.75-100)	100 (84.56-100)
Specificity, % (95% CI)	99.15 (96.97-99.90)	99.16 (97.01-99.90)	98.93 (96.19-99.87)
Positive predictive value, % (95% CI)	93.10 (77.23-99.15)	92.31 (74.87-99.05)	91.67 (73.00-98.97)
Negative predictive value, % (95% CI)	100 (98.44-100)	100 (98.46-100)	100 (98.03-100)
Kappa (95% CI)	0.96 (0.91-1)	0.96 (0.90-1)	0.95 (0.88-1)

### Continuous Photoplethysmographic Detection of Atrial Fibrillation

A total of 171 subjects (mean age 53.23 years, SD 13.58 years), 85 (50%) women, finished 14 days of continuous monitoring with a smart band, resulting in 25,482 valid photoplethysmography waveform signals. These signal results were recorded from11 cases of persistent AF, 20 cases of paroxysmal AF, and 140 cases of sinus rhythm, all of which were confirmed by doctors with clinical data (Figure 1, Table 2).

Hypertension was present in 47 (28%) patients; diabetes mellitus, in 23 (14%) patients; coronary artery disease, in 14 (8%) patients; current smoking, in 24(14%) patients; and current drinking, in 32(19%) patients. The prevalence of other diseases such as heart failure was less than 5%. The median CHA2DS2-VASc (congestive heart failure, hypertension, age ≥75 years [doubled], diabetes mellitus, stroke [doubled], vascular disease, age 65-74 years, female sex) score was 1 (IQR=0.75-2.00), while the median HAS-BLED (hypertension, abnormal renal function, abnormal liver function, stroke, bleeding, labile INR [international normalized ratio], age >65 years, drugs or alcohol) score was 0 (IQR=0.00-1.00; Table 2).

**Figure 2:**
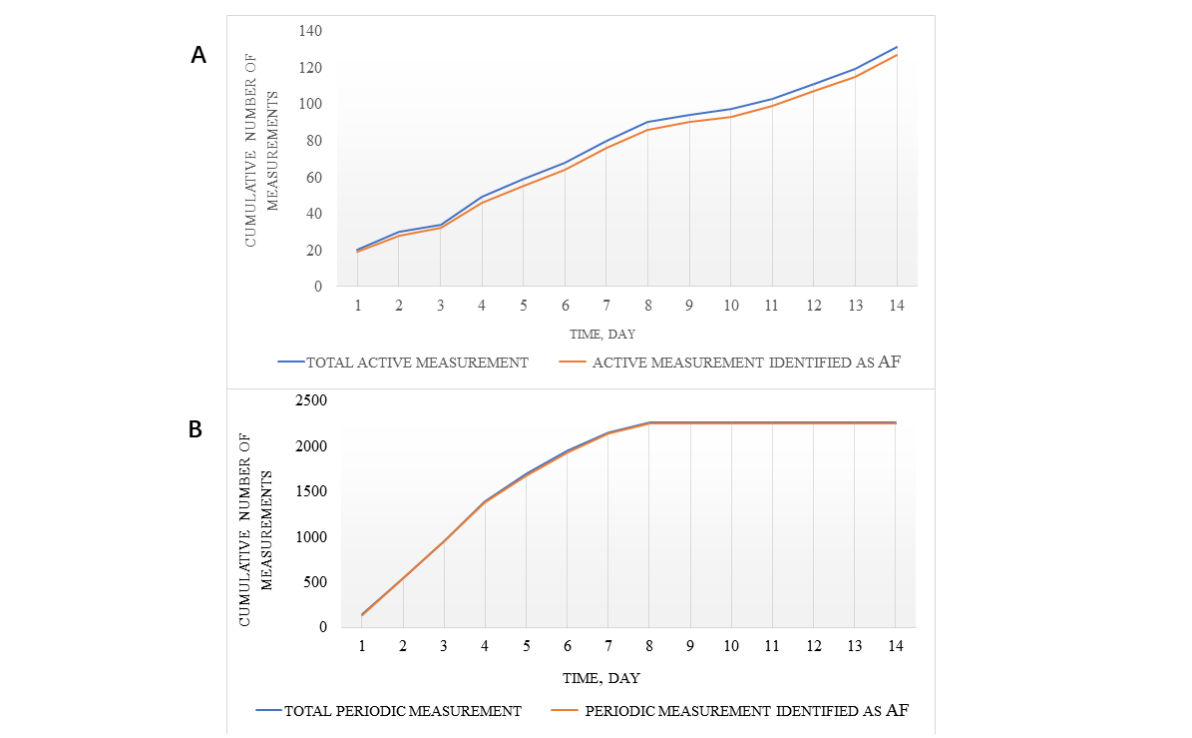
(A) Cumulative active measurement of patients with persistent AF during the 14-day period. (B) Cumulative periodic measurement of patients with persistent AF during the 14-day period. AF: atrial fibrillation.

**Figure 3:**
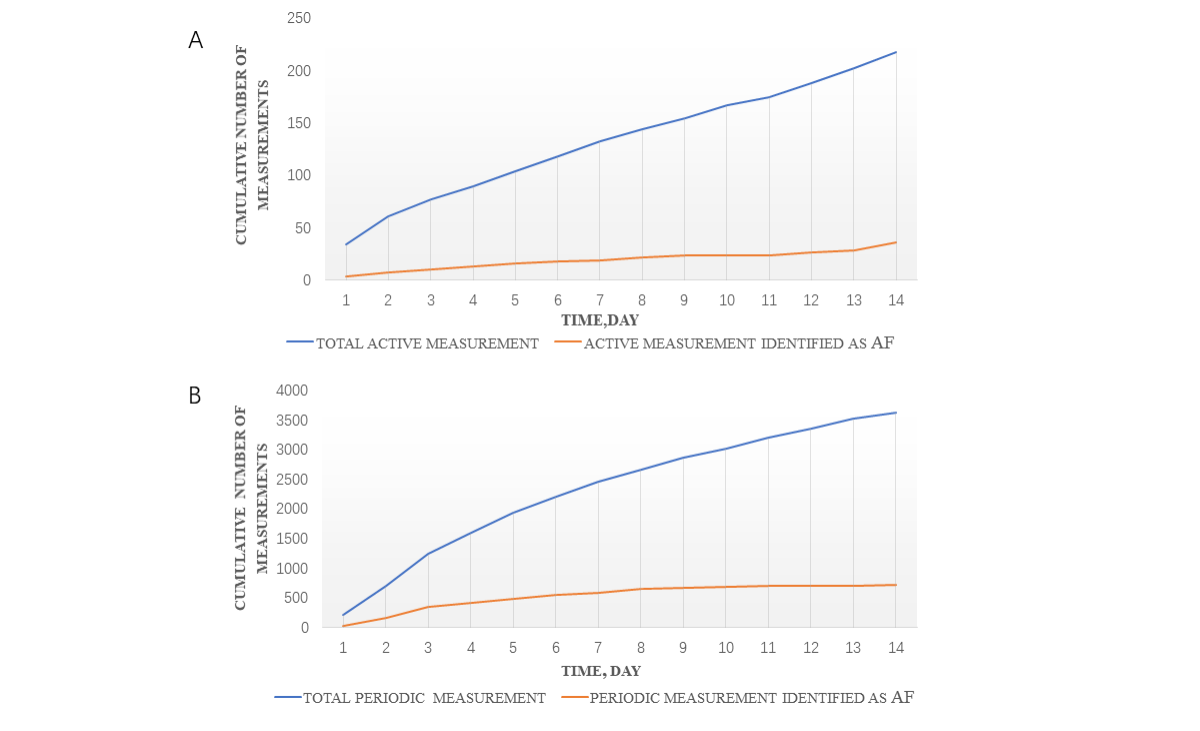
(A) Cumulative active measurement of patients with paroxysmal AF during the 14-day period. (B) Cumulative periodic measurement of patients with paroxysmal AF during the 14-day period. AF: atrial fibrillation.

We finally collected a total of 1618 photoplethysmography active measurement signal segments and 23,864 photoplethysmography periodic measurement signal segments from the 171 participants during the 14-day period (Figure 1). There were 127 (97.0%) active monitoring signals and 2240 (99.2%) periodic monitoring signals suggesting AF during 14 days for patients with persistent AF (Figure 2), while 36 (17%) active monitoring signals and 717 (19.8%) periodic monitoring signals suggested AF for patients with paroxysmal AF (Figure 3). The proportion of AF cumulative episodes to total measurements for 14 days demonstrated significant differences between the persistent AF group and the paroxysmal AF group (periodic measurement: *P*<.001, active measurement: *P*=.001).

The ability of active and periodic measurements to predict AF (median time to first notification of AF) was 1 day (IQR=1.00-1.00) for patients with persistent AF (*P*=.65). For paroxysmal AF, 12 (60%) patients were identified as having AF with periodic measurement and 8 (40%) patients were identified as having AF with active measurement during the 14-day study period, while 14 (70%) patients were identified as having AF within the first 6 days with combined active and periodic measurements (*P*=.15). Median time to first detection of AF was 1 day (IQR=1.00–2.00) and 2 days (IQR=1.25-5.75) by periodic and active measurements, respectively (*P*=.10).

**Table 2:** Baseline characteristics of the continuously monitored participants (N=171).

	Characteristics	Total	
**Demographics**			
	Age (years), mean (SD)	53.23 (13.58)	
	Female, n (%)	85 (50)	
**Medical history**			
	Heart failure, n (%)	1 (1)	
	Hypertension, n (%)	47 (28)	
	Diabetes mellitus, n (%)	23 (14)	
	Previous stroke/SE^a^/TIA^b^, n (%)	4 (2)	
	Coronary artery disease, n (%)	14 (8)	
	Vascular disease, n (%)	8 (5)	
	Renal dysfunction, n (%)	2 (1)	
	Bleeding history or predisposition, n (%)	4 (2)	
	Sleep apnea, n (%)	8 (5)	
	Hyperthyroidism, n (%)	1 (1)	
	Current smoking, n (%)	24 (14)	
	Current drinking, n (%)	32 (19)	
	CHA_2_DS_2_-VASc^c^ score, median (IQR^d^)	1 (0.75-2.00)	
	HAS-BLED^e^ score, median (IQR)	0 (0.00-1.00)	
**Medications, n (%)**			
	Oral anticoagulant	19 (11)	
	Antiplatelet drug	11 (6)	
	Calcium channel blockers	3 (2)	
	ACEI/ARB^f^	10 (6)	
	Diuretic	4 (2)	
	Digoxin	1 (1)	
**Antiarrhythmic drugs, n (%)**			
	Class I	3 (2)	
	Betablocker	3 (2)	
	Class III	5 (3)	
	Class IV	3 (2)	

^a^SE: systemic arterial embolism.

^b^TIA: transient ischemic attack.

^c^CHA2DS2-VASc: congestive heart failure, hypertension, age ≥75 years (doubled), diabetes mellitus, stroke (doubled), vascular disease, age 65-74 years, female sex.

^d^IQR: interquartile range.

^e^HAS-BLED: hypertension, abnormal renal function, abnormal liver function, stroke, bleeding, labile international normalized ratio, age>65 years, drugs or alcohol.

^f^ACEI/ARB: angiotensin-converting-enzyme inhibitor, angiotensin receptor blockers.

### Atrial Fibrillation Burden and Photoplethysmography Monitoring

**Figure 4:**
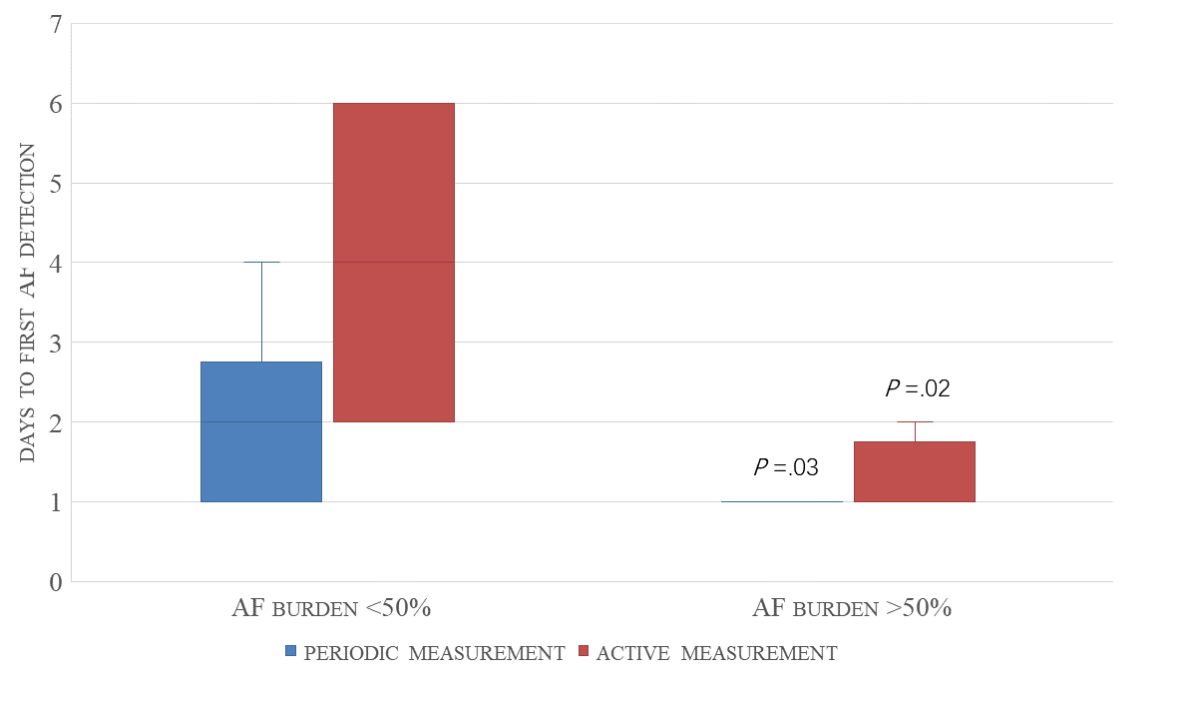
Time to the first detection of AF (quartile box-whisker plot). AF: atrial fibrillation.

AF burden was defined as the ratio of the number of episodes of AF monitored to the total number of measurements for 24 hours. For those with AF episodes sustained less than 50% per 24 hours, the median number of days to first detection of AF was 4 (IQR=2.00-6.00) by active measurement and 2 (IQR=1.00–2.75) by periodic measurement. However, for individuals with AF episodes sustained more than 50% per 24 hours, the median number of days to first detection of AF was 1 for both active (IQR=1.00-1.75) and periodic (IQR=1.00-1.00) measurements. The median number of days to first detection of AF between the two groups demonstrated significant differences (active measurement: *P*=.02, periodic measurement: *P*=.03; Figure 4).

## Discussion

### Principal Findings

This study found that photoplethysmography-based smart devices (watch/band) could effectively detect AF episodes, with active or periodic measurement. However, detection of paroxysmal AF exhibited a longer duration, and therefore, continuous periodic measurement was recommended.

### Accuracy and Sensitivity of Photoplethysmography-Based Smart Device for Detection of Atrial Fibrillation

We analyzed 735 valid photoplethysmography signals acquired by active measuring from 361 participants, excluding 12 subjects with poor pulse signals. Results indicated that the three kinds of smart wearable devices (two watches and one band) exhibited a very high kappa coefficient (0.95-0.96) to detect AF with active measurement against the reference standard diagnosed by doctors. Sensitivity was 100% and specificity ranged from 98.93% to 99.16%, which were higher than the statistics reported by previous works. Tison et al [26] showed that among 51 patients undergoing cardioversion, smart watches based on the photoplethysmography technique can diagnose AF with a sensitivity of 98.0% and a specificity of 90.2% compared to the standard 12-lead electrocardiogram. Rozen et al [27] designed a study to evaluate use of a smartphone app as a method of detecting AF before and after electrical cardioversion in 97 patients and achieved a sensitivity of 93.1% and a specificity of 90.9%.

### Feasibility of Photoplethysmography-Based Smart Device for Continuous 14-Day Monitoring of Atrial Fibrillation

We analyzed a total of 25,482 valid photoplethysmography waveform signals acquired from 171 participants by active and periodic measurements for 14 days. Results showed that periodic measurement can achieve high AF diagnostic accuracy in the real world as well. For patients with persistent AF, 127 (97.0%) and 2240 (99.2%) photoplethysmography signal segments were identified as AF by active and periodic measurements, respectively. These numbers were much higher than those of patients with paroxysmal AF, with only 36 (17%) active monitoring photoplethysmography signals and 717 (19.8%) periodic monitoring photoplethysmography signals suggesting AF. These are consistent with the disease characteristics of persistent and paroxysmal AF; even in patients with persistent AF, there is possibly short sinus rhythm among AF episodes. Periodic measurements seemed to be more sensitive in detecting AF than active measurements. Much frequency monitoring of periodic measurements is likely to “catch” the AF episodes, which also need to be further validated in a large study. In addition, previous studies have shown that 60.3% patients had their first arrhythmia after the first 48 hours of monitoring [28]. In this study, the median time to first AF diagnosis with persistent AF by the two types of measurements were both 1 day. The high accuracy of periodic measurement shows a strong benefit of continuous monitoring. This is important, as patients can not only detect heart arrhythmias at the onset of symptoms such as palpitations via active measuring, as previously mentioned in other studies [29,30], but also improve the identification rate of asymptomatic AF through continuous periodic monitoring.

### Factors Impacting the Continuous 14-Day Detection of Atrial Fibrillation

In a previous study, the mean interval to first detection of AF was inversely proportional to total AF burden [28]. We also compared the time to first detection of AF between different degrees of AF burden. For patients with AF episodes sustained less than 50% in 24 hours, the median number of days to first detection of AF was 4 by active measurement and 2 by periodic measurement. However, the median number of days to first detection of AF was 1 for both measurements for patients presenting with AF episodes sustained more than 50% in 24 hours. This suggested that the less AF occurred, the longer the latency to AF detection (active measurement: *P*=.02, periodic measurement: *P*=.03). Steinhubl et al [31] reported immediate continuous monitoring with a home-based, wearable electrocardiogram sensor patch compared with delayed monitoring, which resulted in a higher rate of AF diagnosis. We found that, with the extension of monitoring time, the number of patients identified with AF gradually increased, especially for patients presenting with paroxysmal AF. As such, paroxysmal AF may require a longer duration of measurement to reach a diagnosis. Moreover, it seems to be a trend that combining active and periodic measurements was more effective in identifying AF than using either of them alone, although this result was not statistically significant. When the two methods were combined, 11 (100%) patients with persistent AF could be detected in the first day. In addition, 14 (70%) patients with paroxysmal AF could be detected within the first 6 days, and no more new patients with AF were detected after 6 days, based on continuous photoplethysmography monitoring data.

### Limitations and Future Directions

There were several limitations to this study. First, the sample size was relatively small. Second, some participants did not complete the active measurement twice a day in strict fidelity with the study requirements. Additionally, the plateau in periodic measurements, resulting from the lack of measurements after the eighth day in patients with persistent AF, could possibly be associated with the discontinuation of smart devices use, which might impact the detection of AF. Third, in 31 (16%) participations diagnosed with sinus rhythm at baseline, AF episodes were detected during the 14-day continuous monitoring. However, these “suspected AF” cases were lost to follow-up, and we could not identify the cardiac rhythm. Fourth, there was no timely 12-lead electrocardiogram monitoring data synchronized with photoplethysmography data in this study. Finally, in 12 (3%) subjects, the recorded quality of photoplethysmography signals was poor. The inadequate signal quality possibly was associated with the deep color, perfusion of the skin, inappropriate wearing, etc. To develop a photoplethysmography-based screening approach, a photoplethysmography algorithm was developed, tested, and optimized among 394 cases in the stage 1 and stage 2. Subsequently, the photoplethysmography algorithm and different smart devices were validated among 375 cases in a real-world settings with 14-day monitoring (stage 3), which was performed in this study. Thereafter, the photoplethysmography algorithm and smart devices would be further validated in the general population with at least 10,000 subjects (stage 4, mAFA II study).

### Conclusions

Photoplethysmography-based smart devices are accurate in the continuous detection of AF outside the hospital. The accuracy is similar between the active and periodic measurements. In addition, this method is simple and accessible. For asymptomatic patients with low AF burden, prolonged continuous monitoring time might increase the detection rate of AF. This technology can extend the diagnosis, monitoring, and risk assessment of AF beyond the hospital, providing a new way for doctors and patients to manage AF together.

## Appendix

Multimedia Appendix 1 Electrocardiogram and photoplethysmography in patients with sinus rhythm.

Multimedia Appendix 2 Electrocardiogram and photoplethysmography in patients with atrial fibrillation.

## Abbreviations

ACEI/ARB: angiotensin-converting-enzyme inhibitor, angiotensin receptor blockers
AF: atrial fibrillation
CHA2DS2-VASc: congestive heart failure, hypertension, age ≥75 years (doubled), diabetes mellitus, stroke (doubled), vascular disease, age 65-74 years, female sex
HAS-BLED: hypertension, abnormal renal function, abnormal liver function, stroke, bleeding, labile international normalized ratio, age>65 years, drugs or alcohol
IQR: interquartile range
SE: systemic arterial embolism
TIA: transient ischemic attack
