# Supplementary figures and images for: Validation of Single Centre Pre-Mobile Atrial Fibrillation Apps for Continuous Monitoring of Atrial Fibrillation in a Real-World Setting: Pilot Cohort Study
Source: J Med Internet Res. 2019 Dec 3;21(12):e14909. doi: 10.2196/14909 (PMC6918204; doi:10.2196/14909)

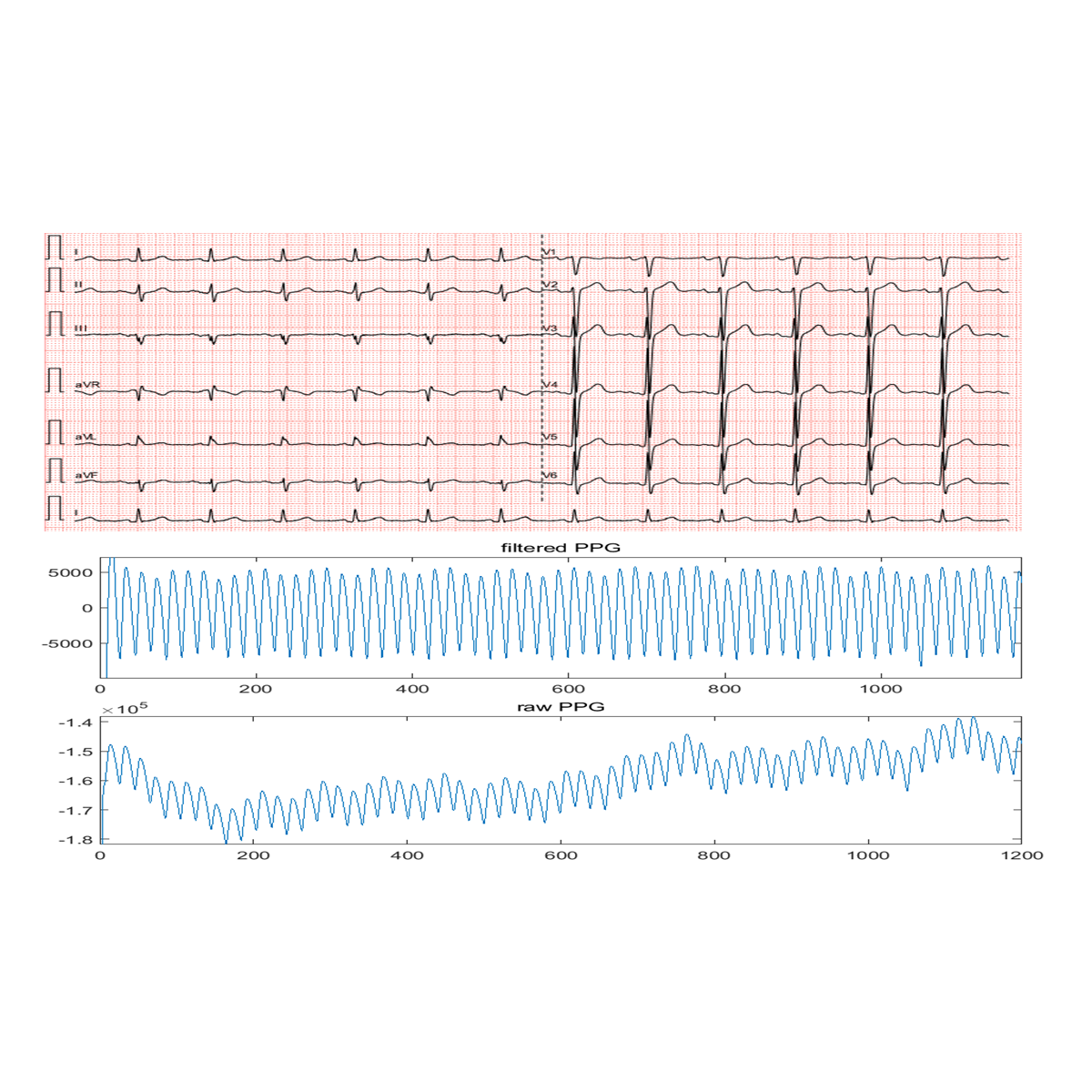

Supplement: Multimedia Appendix 1 [file jmir_v21i12e14909_app1.png]

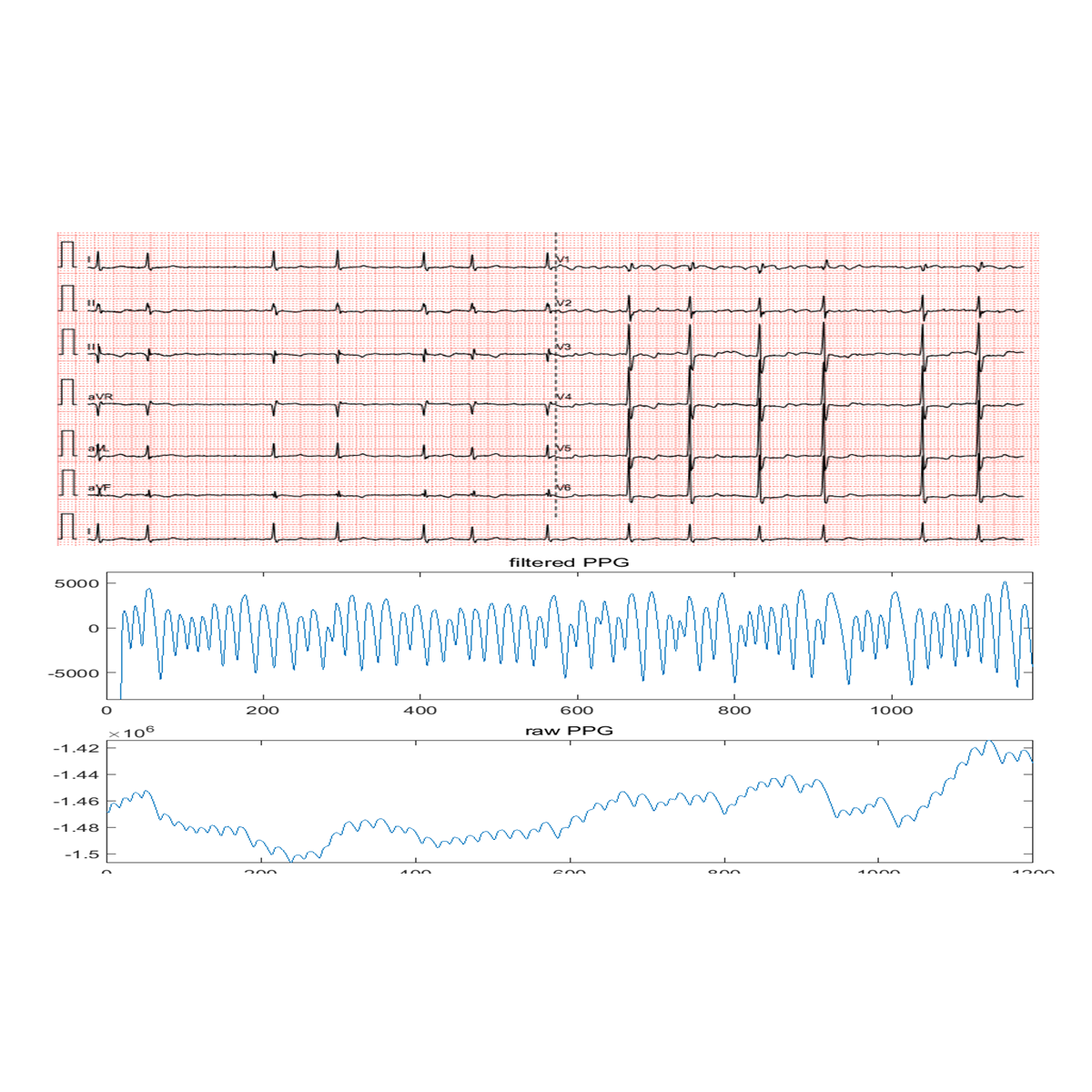

Supplement: Multimedia Appendix 2 [file jmir_v21i12e14909_app2.png]
